# Pseudoprogression as an adverse event of glioblastoma therapy

**DOI:** 10.1002/cam4.1242

**Published:** 2017-11-03

**Authors:** Carmen Balaña, Jaume Capellades, Estela Pineda, Anna Estival, Josep Puig, Sira Domenech, Eugenia Verger, Teresa Pujol, Maria Martinez‐García, Laura Oleaga, JoseMaria Velarde, Carlos Mesia, Rafael Fuentes, Jordi Marruecos, Sonia Del Barco, Salvador Villà, Cristina Carrato, Oscar Gallego, Miguel Gil‐Gil, Jordi Craven‐Bartle, Francesc Alameda, on behalf of the GLIOCAT Group

**Affiliations:** ^1^ Medical Oncology Institut Catala Oncologia (ICO) Badalona Barcelona Spain; ^2^ Radiology Hospital del Mar Barcelona Spain; ^3^ Medical Oncology Hospital Clinic Barcelona Spain; ^4^ Imaging Research Unit Institut de Diagnostic per la Imatge (IDI) Biomedical Research Institute (IDIBGI) Hospital Universitari Dr Josep Trueta Girona Spain; ^5^ Radiology Institut Diagnòstic per la Imatge (IDI) Badalona Barcelona Spain; ^6^ Radiation Oncology Hospital Clinic Barcelona Spain; ^7^ Radiology Hospital Clinic Barcelona Spain; ^8^ Medical Oncology Hospital del Mar Barcelona Spain; ^9^ Statistics Institut Catala Oncologia (ICO) Badalona Barcelona Spain; ^10^ Medical Oncology IDIBELL Institut Catala Oncologia (ICO) Hospitalet de LLobregat Barcelona Spain; ^11^ Radiation Oncology Institut Catala Oncologia (ICO) Girona Spain; ^12^ Medical Oncology Institut Catala Oncologia (ICO) Girona Spain; ^13^ Radiation Oncology, Statistics Institut Catala Oncologia (ICO) Badalona Barcelona Spain; ^14^ Pathology Hospital Germans Trias I Pujol Badalona Barcelona Spain; ^15^ Medical Oncology Hospital de Sant Pau Barcelona Spain; ^16^ Radiation Oncology Hospital de Sant Pau Barcelona Spain; ^17^ Pathology Hospital del Mar Barcelona Spain

**Keywords:** Glioblastoma, IDH1 mutation, imaging, MGMT, pseudoprogression, radionecrosis

## Abstract

We explored predictive factors of pseudoprogression (PsP) and its impact on prognosis in a retrospective series of uniformly treated glioblastoma patients. Patients were classified as having PsP, early progression (eP) or neither (nP). We examined potential associations with clinical, molecular, and basal imaging characteristics and compared overall survival (OS), progression‐free survival (PFS), post‐progression survival (PPS) as well as the relationship between PFS and PPS in the three groups. Of the 256 patients studied, 56 (21.9%) were classified as PsP, 70 (27.3%) as eP, and 130 (50.8%) as nP. Only MGMT methylation status was associated to PsP. MGMT methylated patients had a 3.5‐fold greater possibility of having PsP than eP (OR: 3.48; 95% CI: 1.606–7.564; *P *=* *0.002). OS was longer for PsP than eP patients (18.9 vs. 12.3 months; *P *=* *0.0001) but was similar for PsP and nP patients (*P *=* *0.91). OS was shorter–though not significantly so—for PsP than nP patients (OS: 19.5 vs. 27.9 months; *P *=* *0.63) in methylated patients. PPS was similar for patients having PsP, eP or nP (PPS: 7.2 vs. 5.4 vs. 6.7; *P *=* *0.43). Neurological deterioration occurred in 64.3% of cases at the time they were classified as PsP and in 72.8% of cases of eP (*P *=* *0.14). PsP confounds the evaluation of disease and does not confer a survival advantage in glioblastoma.

## Introduction

Standard first‐line treatment for newly diagnosed glioblastoma is surgery followed by radiotherapy with concomitant and adjuvant temozolomide 1. Pseudoprogression (PsP) is a transient magnetic resonance imaging (MRI) pattern mimicking tumor progression but not necessarily accompanied by clinical deterioration. It occurs most frequently during the first 3 months after radiation therapy and improvement will usually occur within a few weeks or months. PsP is more frequent in patients treated with concomitant temozolomide than in those receiving radiation therapy alone 2, 3 and is particularly frequent in patients with O6‐methylguanine‐DNA methyltransferase (*MGMT*) promoter methylation (MGMT_MET) 4, 5. In spite of new and promising advanced MRI and PET‐based techniques 6, 7, 8, 9, 10, 11, 12, 13, PsP remains indistinguishable from early progression (eP). Since a final diagnosis can only be reached through histopathological verification or subsequent MRIs, PsP can confound treatment monitoring, with direct consequences in clinical practice, as it can lead to prematurely withholding adjuvant temozolomide or to overestimating the efficacy of a second‐line therapy. It is thus recommended to continue with adjuvant temozolomide for at least 3 months after concomitant therapy regardless of findings on the first post‐radiation evaluation. However, some patients experience neurological decline that precludes maintaining adjuvant temozolomide, while patients with true eP need to wait until the following evaluation instead of being switched immediately to another, potentially effective, treatment 2, 14.

There is thus a clear need for imaging, clinical or molecular markers to discriminate PsP from eP. In this study, we retrospectively examined pre‐treatment predictive factors of PsP among glioblastoma patients. In addition, we examined the potential impact of PsP on outcome and compared progression‐free survival (PFS), post‐progression survival (PPS) and overall survival (OS) in patients with PsP, eP, or neither (nP).

## Methods

### Patients

**Figure 1:**
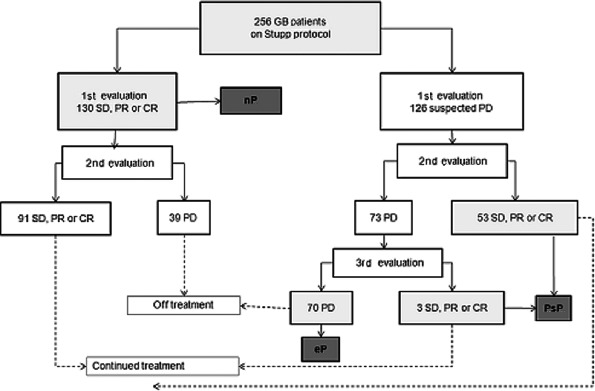
Consort diagram showing flow of patients through the study. SD, stable disease; PR, partial response; CR, complete response; PD, progressive disease; PsP, pseudoprogression; eP, early progression; nP, neither pseudoprogression nor early progression.

Figure 1 shows the flow of patients through the study. Between July 2004 and April 2015, the GLIOCAT project recruited 432 consecutive patients with glioblastoma from six institutions, all of whom had received the standard first‐line treatment. All patients were reviewed histologically to confirm the presence of astrocytic tumor with microvascular proliferation and/or necrosis. The 256 patients who had completed the concurrent treatment with radiotherapy and temozolomide and who had had their first MRI evaluation within 2 months after the completion of radiotherapy were selected for this retrospective substudy. This study was approved by the Ethics Committees of all the participating institutions. The investigators obtained informed consent of each participant or each participant's guardian to be included in the project.

Disease progression was defined as MRI progression and/or clinical deterioration that led to stopping the planned first‐line therapy and changing to either best supportive care or second‐line therapy. Patients were classified in three groups: PsP, eP, or nP. Those who were diagnosed as having suspected progressive disease at their first evaluation but whose subsequent MRI evaluations (performed after the third and sixth cycle of adjuvant temozolomide) showed stable disease or partial or complete response were classified as PsP. Those whose first MRI evaluation showed a pattern indicating suspected progressive disease that was confirmed at subsequent evaluations were classified as eP. Those with progressive disease at the first evaluation, even if based only on neurological deterioration, but who stopped adjuvant temozolomide were also classified as eP. Finally, those whose first evaluation showed stable disease or partial or complete response and who continued treatment with adjuvant temozolomide with no worsening of disease at subsequent evaluations while on this treatment were classified as nP. After the first MRI evaluation, subsequent MRIs were performed after the third and sixth cycle of temozolomide.

The date of progression was the date of the first progression if subsequent MRI evaluations showed no improvement.

### Baseline clinical, molecular, and imaging data

MGMT_MET was assessed by methylation‐specific PCR and isocitrate dehydrogenase 1 (IDH1) mutations by immunohistochemistry. Information was obtained on the date of progression, treatment after progression, and date of death or last control. Tumor imaging characteristics (size, location and eloquence)^15^ were evaluated on axial sections on pre‐surgical MRIs that were available to all radiologists through a shared platform. (See Table 1 for explanation.)

**Table 1:** Characteristics of patients according to clinical factors and subgroup (PsP vs. eP vs. nP)

Characteristic	All patients *N *=* *256 *N* (%)	PsP *N *=* *56 *N* (%)	eP *N *=* *70 *N* (%)	nP *N *=* *130 *N* (%)	*P* a
Age, years					0.59
Median (range)	61 (23–80)	59 (28–77)	62 (40–79)	61 (23–80)	
<50	51 (19.9)	12 (21.4)	11 (17.5)	28 (21.5)	
≥50	205 (80.1)	44 (78.6)	59 (84.3)	102 (78.5)	
Gender					0.37
Male	157 (61.3)	33 (58.9)	39 (55.7)	85 (65.4)	
Female	99 (38.7)	23 (41.4)	31 (44.3)	45 (34.6)	
KPS					0.52
70–100	191 (74.6)	40 (71.4)	50 (71.4)	101 (77.7)	
<70	65 (25.4)	16 (28.6)	20 (28.6)	29 (22.3)	
Anticonvulsant drugs					0.55
No	136 (53.1)	28 (50.0)	41 (58.6)	67 (51.5)	
Yes	120 (46.9)	28 (50.0)	29 (41.4)	63 (48.5)	
Type of surgery					0.06
Complete resection	36 (14.1)	5 (8.9)	5 (7.1)	26 (20.0)	
Partial resection	192 (75.0)	46 (82.1)	58 (82.9)	88 (67.7)	
Biopsy only	28 (10.9)	5 (8.9)	7 (10.0)	16 (12.3)	
Post–op therapy					
Received 60 Gy	253 (98.8)	54 (96.4)	69 (98.6)	130 (100)	0.11
Completed concurrent TMZ	243 (94.9)	54 (96.4)	64 (91.4)	125 (96.2)	0.24
Median no. TMZ cycles (range)	5 (1–20)	6 (2–20)	2 (1–5)	6 (1–12)	–
*MGMT* methylation^b^	(*N *=* *221)	(*N *=* *50)	(*N *=* *66)	(*N *=* *105)	0.005
Methylated	109 (49.3)	34 (68.0)	25 (37.9)	50 (47.6)	
Unmethylated	112 (50.7)	16 (32.0)	41 (62.1)	55 (52.4)	
IDH1 mutations^b^	(*N *=* *162)	(*N *=* *38)	(*N *=* *49)	(*N *=* *75)	0.09
Detected	9 (5.5)	2 (5.3)	0 (0)	7 (9.3)	
Not detected	153 (94.5)	36 (94.7)	49 (100)	68 (90.7)	
MMSE score	(*N* = 135)	(*N* = 32)	(*N* = 27)	(*N* = 76)	
<27	49 (19.1)	15 (46.9)	10 (37.0)	24 (31.6)	
≥27	86 (33.6)	17 (53.1)	17 (63.0)	52 (68.4)	0.32
Dexamethasone dose at start of concurrent therapy	(*N* = 234)	(*N* = 53)	(*N* = 63)	(*N* = 118)	0.01
≤2 mg	63 (26.9)	10 (18.9)	11 (17.5)	42 (35.6)	
>2 mg	171 (73.1)	43 (81.1)	52 (82.5)	76 (64.4)	
Dexamethasone stopped during concurrent therapy					0.60
No	222 (86.7)	50 (89.3)	62 (88.6)	110 (84.6)	
Yes	34 (13.3)	6 (10.7)	8 (11.4)	20 (15.4)	
Tumor size T1 Gd^c^	(*N* = 194)^c^	(*N* = 41)^c^	(*N* = 49)^c^	(*N* = 104)^c^	0.30
≤5 cm	103 (53.1)	25 (61.0)	28 (57.1)	50 (48.1)	
>5 cm	91 (46.9)	16 (39.0)	21 (42.9)	54 (51.9)	
Tumor size T2 Flair^c^	(*N* = 185)^c^	(*N* = 39)^c^	(*N* = 46)^c^	(*N* = 103) c	0.91
≤5 cm	32 (17.3)	7 (17.9)	7 (15.2)	18 (18.0)	
>5 cm	153 (82.7)	32 (82.1)	39 (84.8)	85 (82.0)	
Tumor location T1 Gd^c^	(*N* = 194)^c^	(*N* = 41)^c^	(*N* = 49)^c^	(*N* = 104)^c^	0.11
Group A	103 (53.1)	18 (43.9)	22 (44.9)	63 (60.6)	
Group B	72 (37.1)	17 (41.5)	24 (49.0)	31 (29.8)	
Group C	19 (9.8)	6 (14.6)	3 (61.1)	10 (9.6)	
Tumor lo^c^ation T2 Flair^c^	(*N* = 184)^c^	(*N* = 40)^c^	(*N* = 56)^c^	(*N* = 98)^c^	0.35
Group A	87 (47.3)	17 (42.5)	17 (37.0)	53 (54.1)	
Group B	65 (35.3)	16 (40.0)	20 (43.5)	29 (29.6)	
Group C	32 (17.4)	7 (17.5)	9 (19.6)	16 (16.3)	
Eloquence T1 Gd^c^	(*N* = 193)^c^	(*N* = 41)^c^	(*N* = 48)^c^	(*N* = 104)^c^	0.16
No	138 (71.5)	28 (68.3)	30 (62.5)	80 (76.9)	
Yes	55 (28.5)	13 (31.7)	18 (37.5)	24 (23.1)	
Eloquence T2 Flair^c^	(*N* = 188)^c^	(*N* = 40)^c^	(*N* = 47)^c^	(*N* = 101)^c^	0.11
No	124 (66.0)	25 (62.5)	26 (55.3)	73 (72.3)	
Yes	64 (34.0)	15 (37.5)	21 (44.7)	28 (27.7)	

PsP, pseudoprogression; eP, early progression; nP, neither pseudoprogression nor early progression; KPS, Karnofsky performance status; TMZ, temozolomide; MMSE, Mini Mental State Examination. Percentages are given over the number of patients with available data.

a *P*‐values are given for comparison of the distribution of variables in the three subgroups of patients (PsP vs. eP v.s nP). The analyses comparing the distribution of variables between PsP and eP showed no significant differences between the groups except for MGMT methylation (*P *=* *0.001).

b Among the 109 patients with MGMT methylation, 34 (31.2%) were classified as PsP, 25 (22.9%) as eP, and 50 (45.9%) as nP. Of the 112 patients without MGMT methylation, 16 (14.3%) were classified as PsP, 41 (36.6%) as eP, and 55 (52.4%) as nP. Among patients with PsP, 68% had MGMT methylation. Five patients were secondary GBM and had had previous surgery for low‐grade glioma. Only two of these patients had IDH1‐mutated tumors. Seven patients had IDH1‐mutated tumors without evidence of a previous diagnosis of a lower grade glioma.

c Tumor size was measured on T1 enhanced sequences and on T2 Flair images. In the case of multiple enhanced lesions, the sum of the greatest diameters was estimated as a variable. Location was evaluated in three groups: group A tumors were located in the right cerebral hemisphere or left occipital lobe; group B tumors were located in the left frontal, parietal or temporal lobe; and group C tumors were located mainly in or partly extending to the thalamus, caudate nucleus and/or internal capsule. Tumors partly extending to the structures medial to the internal capsule, even if located mainly outside the midline structures, were classified as group C, together with other deep‐seated tumors and were considered as eloquent areas. Tumors in eloquent brain areas were those located in the sensorimotor cortex, language cortex, internal capsule, thalamus, corpus callosum, fornix, hypothalamus, and brain stem.

### Statistical analyses

Categorical variables were compared with the *χ* ^2^ or Fisher's exact test. PFS was defined as the time from surgery to documented disease progression or death from any cause. PPS was defined as the time from confirmed progression to the last visit or death. OS was defined as the time from surgery to death from any cause. Patients who were still progression‐free or alive at the date of last contact were censored. Median PFS, PPS and OS were calculated with the Kaplan–Meier method and compared using the log‐rank test. The Cox proportional hazards model was used to calculate hazard ratios (HRs) with their 95% confidence intervals (CIs). The non‐parametric Kruskall–Wallis test or the Mann–Whitney *U* test, as appropriate, was used to compare the PFS‐PPS relationship among the patient groups. All statistical tests were two‐sided and significance was set at 0.05. All analyses were performed with SPSS v24.0 (IBM).

### Literature search criteria

We performed an NCBI Pubmed search for articles published in English before March 2017 to identify publications on Pseudoprogression or Radionecrosis. Using the key words “pseudoprogression AND glioblastoma’’ and “radionecrosis AND glioblastoma’’, we had 770 and 220 hits, respectively. We then selected publications reporting data on patients and reviews that focused on “imaging”, “MGMT”, “IDH mutations”, “surgery”, “clinical characteristics”, “clinical outcome”, and “differential diagnosis”. Finally, we selected 130 publications for review and cited the ones most relevant to our study in our manuscript.

## Results

Patient characteristics are shown in Table 1. Fifty‐six patients (21.9%) were classified as PsP, 70 (27.3%) as eP, and 130 (50.8%) as nP (Fig. 1).

There were significant differences in MGMT_MET status among the three patient groups (*P *=* *0.005), between PsP and eP patients (*P *=* *0.001), and between PsP and nP patients (*P *=* *0.02). Patients with MGMT_MET had a 3.5‐fold greater possibility of having PsP rather than eP (odds ratio [OR]: 3.48; 95% CI: 1.606–7.564; *P *=* *0.002).

Significant differences in dexamethasone dose (*P *=* *0.01) and a trend toward differences in type of surgery (*P *=* *0.06) and in IDH1 mutation status (*P *=* *0.09) were also observed among the three patient groups. There were no differences among the three patient groups of patients in tumor size, tumor location or the eloquence of adjacent brain, as measured by T1 Gd or T2 Flair sequences (details in Table 1).

### Overall survival

With a median follow‐up of 17.0 months (range: 10.7–24.5), age ≤50 years, complete resection, MGMT_MET, and IDH1 mutations were associated with longer OS. Patients with eP had shorter OS (12.3 months) than those with PsP (18.9 months) or nP (19.7 months) (*P *<* *0.001). There were no significant differences between those with PsP and those with nP (*P *=* *0.91).

Among patients with MGMT_MET, those with nP had longer OS than those with PsP, although this difference was not significant (27.9 vs. 19.5 months). Among patients without MGMT_MET, OS was similar for patients with PsP and those with nP (17.9 vs. 17.5 months) (Table S1).

### Post‐progression treatment and PPS

At the time of analysis, 245 patients had relapsed after first‐line treatment. Treatments at relapse, including hospice care, were similar across the three patient subgroups. Neurological deterioration—either alone or with MRI progression—occurred in 64.3% of cases at the time they were classified as PsP and in 72.8% of cases of eP, while imaging progression with no neurological deterioration was observed in 30.4% of patients with PsP and in 22.9% of those with eP (*P *=* *0.02) (Table S2).

After progression, PPS was similar for all patients whatever response to first‐line therapy (*P *=* *0.43) or methylation status were (*P *=* *0.35) (Figure S1). Among patients with MGMT_MET, PPS was 7.9 months for those with PsP, 3.1 months for those with eP, and 7.3 months for those with nP (*P *=* *0.17). PsP was not better than nP regarding PFS (*P *=* *0.81) or PPS (*P *=* *0.92) even when analyzed by MGMT status (Table 2).

**Table 2:** Progression‐free survival and post‐progression survival

	*N*	PFS (mo)	95% CI	*P*	*N*	PPS (mo)	95% CI	*P*
All patients	256	8.5	8.1–9.0		245	6.7	5.4–8.1	
PsP vs. all other patients				0.01				0.78
PsP	56	10.5	10.0–11.1		53	7.2	5.2–9.2	
eP + nP	200	7.8	7.2–8.5		192	6.7	4.9–8.6	
PsP vs. nP				0.81				0.92
PsP	56	10.5	10.0–11.1		53	7.2	5.2–9.2	
nP	130	10.4	9.0–11.8		122	6.7	5.0–8.6	
PsP vs. eP vs. nP				0.0001				0.43
PsP	56	10.5	10.0–11.1		53	7.2	5.2–9.2	
eP	70	5.3	5.0–5.7		70	5.4	1.0–9.9	
nP	130	10.4	9.0–11.8		122	6.7	5.0–8.6	
MGMT status				0.0001				0.35
Methylated	109	8.8	7.3–10.3		101	6.7	5.0–8.5	
Unmethylated	112	8.2	7.4–9.0		109	7.0	4.7–9.3	
Subanalysis among patients with MGMT methylation								
PsP vs. nP				0.35				0.96
PsP	34	10.3	6.9–13.7		33	7.9	6.0–9.9	
nP	50	13.9	8.8–19.0		43	7.3	4.4–10.2	
PsP vs. eP vs. nP				0.0001				0.17
PsP	34	10.3	6.9–13.7		33	7.9	6.0–9.9	
eP	25	5.5	5.0–6.1		25	3.1	0.8–5.6	
nP	50	13.9	8.8–19.0		43	7.3	4.4–10.2	
Subanalysis among patients without MGMT methylation								
PsP vs. nP				0.23				0.67
PsP	16	10.5	10.1–11.0		14	6.4	1.2–11.7	
nP	55	9.7	7.9–11.5		54	6.8	3.5–10.1	
PsP vs. eP vs. nP				0.0001				0.71
PsP	16	10.5	10.1–11.0		14	6.4	1.2–11.7	
eP	41	5.5	4.9–6.1		41	7.8	4.3–11.4	
nP	55	9.7	7.9–11.5		54	6.8	3.5–10.1	

PPS, post‐progression survival; PsP, pseudoprogression; PFS, progression‐free survival

**Figure 2:**
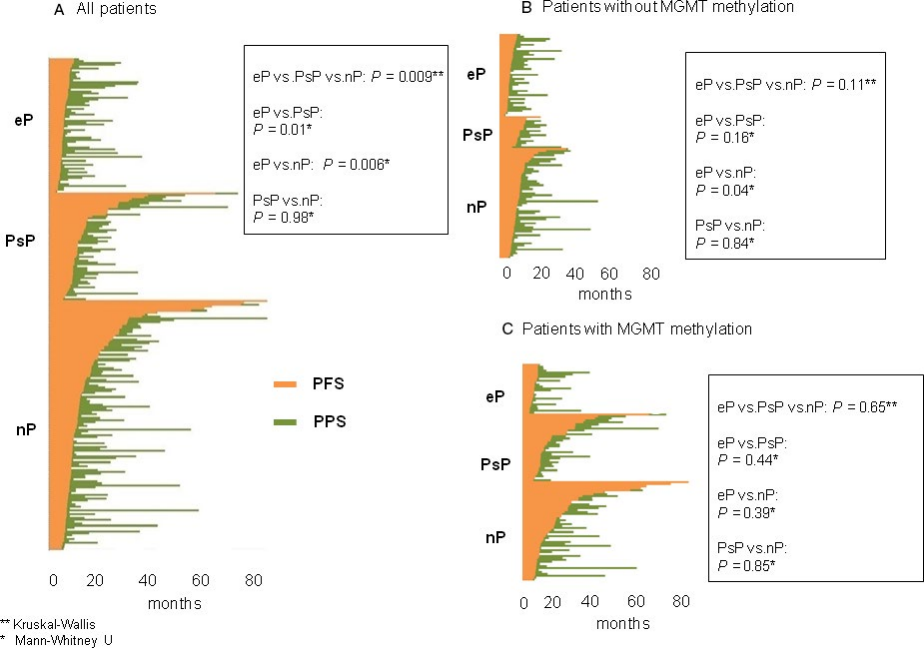
Relationship between PFS and PPS in (A) all patients, (B) patients without MGMT methylation (MGMT_MET), and (C) patients with MGMT_MET. *P*‐values are given for the comparison of the relationship between PFS and PPS. pfs, progression‐free survival; PPS, post‐progression survival

Some patients classified as eP had long PPS after second‐line therapy (Fig. 2A–C). Though it seemed logical to hypothesize that patients with shorter PFS would also have shorter PPS, this was not always the case. Differences in this pattern were significant when comparing PsP versus eP versus nP (*P *=* *0.009), PsP versus eP (*P *=* *0.01), and eP versus nP (*P *=* *0.006), but not when comparing PsP and nP. Among patients without MGMT_MET, these differences were maintained to a certain extent for eP versus nP (*P *=* *0.04) but not in other cases. Among patients with MGMT_MET, there were no differences between patients classified as eP and those classified as PsP or nP.

## Discussion

We have reviewed our series of patients in order to identify predictive factors for PsP and found that MGMT_MET patients had a 3.5‐fold greater possibility of having PsP than eP. In consistent with previous reports 4, 5, 16, 17, none of the IDH1‐mutated patients had eP (see Table 1)and patients undergoing gross total resection showed a trend toward a lower probability of having either PsP or eP. To the best of our knowledge, this is the first study to examine the potential association between imaging baseline tumor characteristics (size, location or eloquence) and PsP. However, none of these characteristics was associated with either PsP or eP (Table 1).

Previous studies have reported PsP in 12‐50% of patients 18, 19, 20, 21 although when applying only strict MRI imaging criteria, the incidence can be as low as 11.4% 22. Neurological decline accompanied PsP in 64.3% of our patients and was not useful for distinguishing PsP and eP. Since neurological decline takes months to stabilize and may preclude continuing with temozolomide, it is likely a major reason for the high interest in PsP 23.

Our findings on outcome confirm previous reports 4, 19, 20, 23 that patients with PsP have longer OS than those with eP but not than those with nP.

Confounding factors in the evaluation of progression are PsP, late PsP and radionecrosis. Although radionecrosis is considered a late adverse event 24, 25, it can also overlap in time with PsP. PsP has been observed later than the first 3 months post‐radiotherapy 10, 18, 22, 26 and radionecrosis has been found in surgical specimens immediately after radiotherapy 27, 28. Radionecrosis is pathologically defined 24 and a mixed pattern of viable tumor and radionecrosis can be found in PsP 29, 30. Clinically, both PsP and radionecrosis can be accompanied by cognitive and neurological decline that can stabilize or reverse 2, 4, 23, 31. The primary risk factor for radionecrosis is total radiation dose, fraction size and irradiated volume; it rarely appears at doses lower than 50 Gy and standard fractions of 1.8–2.0 Gy 30, 32. Chemotherapy increases the risk of both radionecrosis and PsP 25. The higher incidence of PsP in patients with MGMT_MET could be explained by the rationale for combining temozolomide with radiotherapy, based on preclinical data suggesting additive or synergistic activity 33.

A limitation of the study is that our findings are based on retrospective data not subjected to a pre‐specified periodicity of disease evaluations. The only prospective study published in 2008 did not compare post‐progression outcomes and did not take into account late PsP or radionecrosis as confounding factors 4. Our study brings up the current existing dilemma when evaluating the disease: some of our patients classified as eP who had relatively long PPS on second‐line therapy could well have had a PsP, and those who progressed later than 3 months post‐radiotherapy could well have had late PsP or radionecrosis 10, 22, 26 since no pathological diagnosis was obtained. All these confounding factors may explain the difference in the patterns shown in Figure 2, especially considering the limited efficacy of treatments for recurrent disease.

According to our results, PsP (and radionecrosis) should be considered confounding factors and undesired adverse events of concomitant therapy rather than indications of treatment efficacy. Consequently, we suggest they should be reported as such in clinical trials exploring alternative radiotherapy doses and fractions combined with chemotherapy 34, 35, 36. Moreover, we could speculate that it would also be of interest to investigate a reduction in total radiation dose to reduce the risk of PsP especially in patients with MGMT_MET.

## Conflict of Interest

Authors state they do not have any conflict of interest.

## Supporting information


**Table S1.** Overall survival according to clinical and molecular characteristics.

**Table S2.** Type of progression and second‐line therapies according to subgroup of patients (PsP vs. eP vs. nP).

**Figure S1.** Post‐progression survival for patients classified as PsP, eP, or nP.
