# Supplementary material for: Pseudoprogression as an adverse event of glioblastoma therapy
Source: Cancer Med. 2017 Nov 3;6(12):2858–66. doi: 10.1002/cam4.1242 (PMC5727237; doi:10.1002/cam4.1242)
Supplement: Supplementary file 1 — Table S1. Overall survival according to clinical and molecular characteristics. [file CAM4-6-2858-s001.docx]

**Supplementary Table S1:** Overall Survival According to Clinical and Molecular Characteristics.

| **Variable** | ***N*** | | **OS (mos)** | | **95% CI** | ***P*** | **HR** | **95% CI** | ***P*** |
| --- | --- | --- | --- | --- | --- | --- | --- | --- | --- |
| **Total** | 256 | | 17.6 | | 15.92-19.41 |  |  |  |  |
| **Age**  ≤50  >50 | 51  205 | | 21.46  16.36 | | 18.71-24.22  14.14-18.58 | 0.006 | 0.622 | 0.443-0.875 | 0.006 |
| **KPS**  ≥70  <70 | 191  65 | | 18.03  14.76 | | 16.19-19.87  9.95-19.57 | 0.68 | 0.937 | 0.688-1.276 | 0.68 |
| **MMSE score**  <27  ≥27 | 49  86 | | 15.0  21.1 | | 11.38-18.68  18.82-23.72 | 0.20 | 1.284 | 0.877-1.880 | 0.20 |
| **Type of surgery**  Complete resection  Partial resection  Biopsy only | 36  181  28 | | 23.3  17.1  13.1 | | 17.10-29.49  15.21-19.05  6.66-19.53 | 0.004 | 1.590 | 1.198-2.110 | 0.001 |
| **MGMT methylation**  Methylated  Unmethylated | 109  112 | | 20.6  15.5 | | 16.65-24.67  13.66-17.40 | 0.0001 | 0.571 | 0.424-0.770 | 0.0001 |
| **IDH1 mutations**  Detected  Not detected | 9  153 | | 33.56  17.12 | | 25.87-41.26  15.22-19.04 | 0.006 | 0.333 | 0.145-0.761 | 0.009 |
| **Patient sub-groups**  PsP eP  nP | 56  70  130 | | 18.9  12.3  19.7 | | 15.55-22.38  10.29-14.43  17.28-22.24 | 0.0001 | 1.548 | 1.312-1.816 | 0.0001 |
| **Patient sub-groups**  PsP eP | 56  70 | | 18.9  12.3 | | 15.55-22.38  10.29-14.43 | 0.0001 | 2.432 | 1.637-3.614 | 0.0001 |
| **Patient sub-groups**  PsP  nP | 56  130 | | 18.9  19.7 | | 15.55-22.38  17.28-22.24 | 0.91 | 0.980 | 0.692-1.389 | 0.91 |
| **Sub-analysis of overall survival in patients with MGMT methylation** | | | | | | | | | |
| PsP  nP | 34  50 | 19.5  27.9 | | 13.2-25.8  18.7-37.2 | | 0.63 | 0.884 | 0.533-1.468 | 0.64 |
| **Sub-analysis of overall survival in patients without MGMT methylation** | | | | | | | | | |
| PsP  nP | 16  55 | 17.9  17.5 | | 14.0-21.8  15.2-19.7 | | 0.82 | 0.935 | 0.519-1.684 | 0.82 |

OS, overall survival; HR, hazard ratio; KPS, Karnofsky performance status; MMSE, Mini Mental State Examination
