# Supplementary material for: Pseudoprogression as an adverse event of glioblastoma therapy
Source: Cancer Med. 2017 Nov 3;6(12):2858–66. doi: 10.1002/cam4.1242 (PMC5727237; doi:10.1002/cam4.1242)
Supplement: Supplementary file 2 — Table S2. Type of progression and second‐line therapies according to subgroup of patients (PsP vs. eP vs. nP). [file CAM4-6-2858-s002.docx]

**Supplementary Table S2:** Type of progression and second-line therapies according to sub-group of patients (PsP vs. eP vs. nP).

|  | **PsP**  ***N*=56**  ***N* (%)** | **eP**  ***N*=70**  ***N* (%)** | **nP**  ***N*=130**  ***N* (%)** |
| --- | --- | --- | --- |
| **Type of progression^a^** | | | |
| Radiological progression only | 17 (30.4) | 16 (22.9) | 49 (37.7) |
| Radiological progression + neurological deterioration | 27 (48.2) | 47 (67.1) | 55 (42.3) |
| Neurologic deterioration only | 9 (16.1) | 4 (5.7) | 15 (11.5) |
| No progression | - |  | 8 (6.2) |
| No data | 3 (5.4) | 3 (4.3) | 3 (2.3) |
| **Treatment at progression** | | | |
| Patients with progression | 53 | 70 | 122 |
| Surgery +/- chemotherapy or re-irradiation | 12 (22.7) | 4 (5.7) | 21 (17.2) |
| Systemic treatment | 26 (49.1) | 37 (52.9) | 64 (52.5) |
| Nitrosoureas or temozolomide | 10 (38.4) | 9 (24.3) | 23 (36.0) |
| Bevacizumab +/- irinotecan | 15 (57.7) | 21 (56.7) | 29 (45.3) |
| Clinical trial | 1 (3.9) | 7 (19.0) | 12 (18.7) |
| No further treatment**^b^** | 15 (28.3) | 26 (37.1) | 35 (28.7) |
| Unknown^c^ | - | 3 (4.3) | 2 (1.6) |

**^a^** At the time of PsP or eP or when nP progressed. Differences in type of progression (radiological, neurological, or both) were observed between patients classified as PsP and those classified as eP (*P* = 0.05) although the frequency of neurological deterioration with or without radiological progression was similar for patients with PsP and those with eP (*P* = 0.14). Patients classified as nP had a higher frequency of radiological progression without neurological deterioration than those classified as eP (*P* = 0.02).

**^b^** Percentages over the patients who progressed. There were no differences in the percentage of patients who received no further treatment after progression – either among the three groups (*P* = 0.33) or between PsP and eP (*P* = 0.22).

^c^Some patients were lost to follow-up after progression.
