# Supplementary figures and images for: Pseudoprogression as an adverse event of glioblastoma therapy
Source: Cancer Med. 2017 Nov 3;6(12):2858–66. doi: 10.1002/cam4.1242 (PMC5727237; doi:10.1002/cam4.1242)

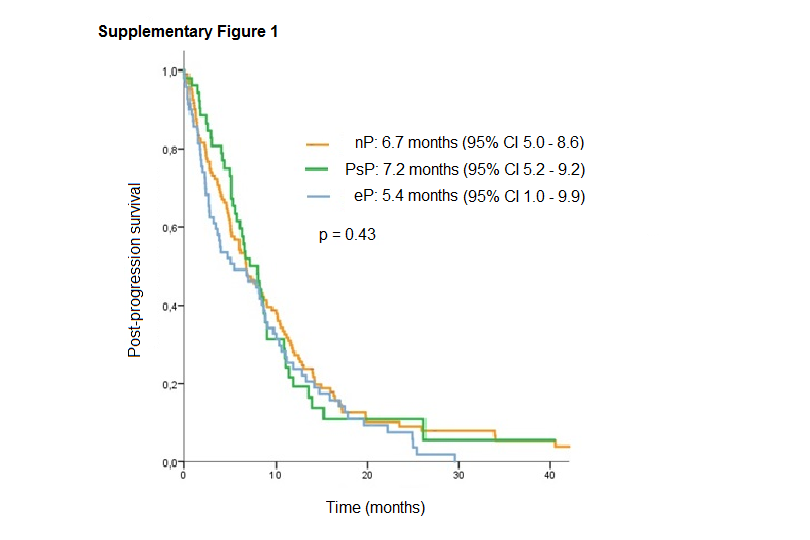

Supplement: Supplementary file 3 — Figure S1. Post‐progression survival for patients classified as PsP, eP, or nP. [file CAM4-6-2858-s003.png]
